# Comparison of the CO-RADS and the RSNA chest CT classification system concerning sensitivity and reliability for the diagnosis of COVID-19 pneumonia

**DOI:** 10.1186/s13244-021-00998-4

**Published:** 2021-04-28

**Authors:** Mohamed Abdel-Tawab, Mohammad Abd Alkhalik Basha, Ibrahim A. I. Mohamed, Hamdy M. Ibrahim, Mohamed M. A. Zaitoun, Saeed Bakry Elsayed, Nader E. M. Mahmoud, Ahmed A. El Sammak, Hala Y. Yousef, Sameh Abdelaziz Aly, Hamada M. Khater, Walid Mosallam, Waleed S. Abo Shanab, Ali M. Hendi, Sayed Hassan

**Affiliations:** 1 Department of Diagnostic Radiology, Faculty of Human Medicine, Assiut University, Assiut, Egypt; 2 Department of Diagnostic Radiology, Faculty of Human Medicine, Zagazig University, Zagazig, Egypt; 3 Department of Radio-diagnosis, Faculty of Human Medicine, Benha University, Benha, Egypt; 4 Department of Radio-diagnosis, Faculty of Human Medicine, Suiz Canal University, Esmaelia, Egypt; 5 Department of Radio-diagnosis, Faculty of Human Medicine, Port Said University, Port Said, Egypt; 6 Department of Radiology, College of Medicine, Jazan University, Jazan, Saudi Arabia

**Keywords:** COVID-19, Pneumonia, Tomography (X-ray computed), Reproducibility of results, Retrospective studies

## Abstract

**Background:**

The Radiological Society of North America (RSNA) recently published a chest CT classification system and Dutch Association for Radiology has announced Coronavirus disease 2019 (COVID-19) reporting and data system (CO-RADS) to provide guidelines to radiologists who interpret chest CT images of patients with suspected COVID-19 pneumonia. This study aimed to compare CO-RADS and RSNA classification with respect to their sensitivity and reliability for diagnosis of COVID-19 pneumonia.

**Results:**

A retrospective study assessed consecutive CT chest imaging of 359 COVID-19-positive patients. Three experienced radiologists who were aware of the final diagnosis of all patients, independently categorized each patient according to CO-RADS and RSNA classification. RT-PCR test performed within one week of chest CT scan was used as a reference standard for calculating sensitivity of each system. Kappa statistics and intraclass correlation coefficient were used to assess reliability of each system. The study group included 359 patients (180 men, 179 women; mean age, 45 ± 16.9 years). Considering combination of CO-RADS 3, 4 and 5 and combination of typical and indeterminate RSNA categories as positive predictors for COVID-19 diagnosis, the overall sensitivity was the same for both classification systems (72.7%). Applying both systems in moderate and severe/critically ill patients resulted in a significant increase in sensitivity (94.7% and 97.8%, respectively). The overall inter-reviewer agreement was excellent for CO-RADS (*κ* = 0.801), and good for RSNA classification (*κ* = 0.781).

**Conclusion:**

CO-RADS and RSNA chest CT classification systems are comparable in diagnosis of COVID-19 pneumonia with similar sensitivity and reliability.

## Key points

- CO-RADS and RSNA chest CT classification systems are comparable in diagnosis of COVID-19 pneumonia with similar sensitivity and reliability.
- Considering combination of CO-RADS 3, 4 and 5 and combination of typical and indeterminate RSNA categories as positive predictors for COVID-19 diagnosis, the overall sensitivity was the same for both classification systems (72.7%).
- Applying both systems in moderate and severe/critically ill patients resulted in a significant increase in sensitivity (94.7% and 97.8%, respectively).
- The overall inter-reviewer agreement was excellent for CO-RADS (*κ* = 0.801) and good for the RSNA classification (*κ* = 0.781).
- The CO-RADS had a better inter-reviewer agreement that may be attributed to greater familiarity with the CO-RADS system among radiologists due to its resemblance to other RAD systems.

## Background

Coronavirus disease 2019 (COVID-19) is an acute infectious disease caused by a new strain of coronavirus known as severe acute respiratory syndrome coronavirus 2 (SARS-CoV-2) [1]. The worldwide emergence of this novel virus was declared a pandemic on March 11, 2020 by the World Health Organization and, since then, the world has been struggling to control its spread [2]. Among other methods, accurate, fast diagnostic testing is necessary to prevent potential viral dissemination and to reduce the disease fatality rate [3, 4].

Real-time reverse-transcriptase polymerase chain reaction (RT-PCR) is considered the current gold-standard assessment for the diagnosis of COVID-19 [5]. However, RT-PCR is reported to have a low sensitivity with a considerable number of false-negative results, possibly necessitating that multiple tests be performed even up to five times to exclude the disease, despite the shortage of test kits in many regions all over the world [4, 6]. Moreover, it may take hours or even days for RT-PCR test results to be available [7, 8].

The full availability of CT machines and the short examination time make CT an ideal modality to take on an emerging role in the management of COVID-19 patients and to even act as an excellent alternative to RT-PCR in some circumstances [8], especially in countries with limited availability of RT-PCR testing [2]. CT could differentiate COVID-19 from other lung infections, especially viral ones [9]. Another advantage of CT is its ability to assess disease severity and progression [3, 10] as the volume of pneumonic involvement of the entire lung can suggest both [10, 11]. While the seventh Chinese Novel Coronavirus Pneumonia Diagnosis and Treatment Plan included chest CT imaging in the clinical diagnosis of patients with potential SARS-CoV-2 exposure, the American College of Radiology (ACR) has not recommended CT chest imaging for the initial diagnosis of patients suspected to have COVID-19, leaving it instead only indicated for specific situations [12, 13].

Several trials have been conducted to date to ascertain the proper and standardized reporting style of CT chest image findings in patients with suspected COVID-19 pulmonary involvement. The Radiological Society of North America (RSNA) chest CT classification system includes four categories: negative for pneumonia, atypical, indeterminate, and typical [14]. Another scoring system, the COVID-19 Reporting and Data System (CO-RADS) was developed by the Dutch Association for Radiology with grades ranging from 1 to 5 to suggest ascending disease probability according to the CT chest findings [15]. COVID-19 imaging reporting and data system (COVID-RADS) [16] is another described reporting system; however, it is less widely used.

For the application of any new classification system, it is essential to evaluate its validity and reliability. Few studies to date have been performed to establish the true value of the aforementioned systems as useful, reliable classification systems of chest CT examination findings in patients suspected to have COVID-19. The purpose of this study, therefore, was to compare CO-RADS and the RSNA chest CT classification system with respect to their sensitivity and reliability for the diagnosis of COVID-19 pneumonia.

## Materials and methods

### Ethical statement

The institutional review board of Faculty of Human Medicine, Assiut University approved this study (approval no. 17300425; approved June 7, 2020) and waived the need to gather patients’ formal consent. The study was conducted according to the ethical principles of the Declaration of Helsinki.

### Patient population

**Fig. 1:**
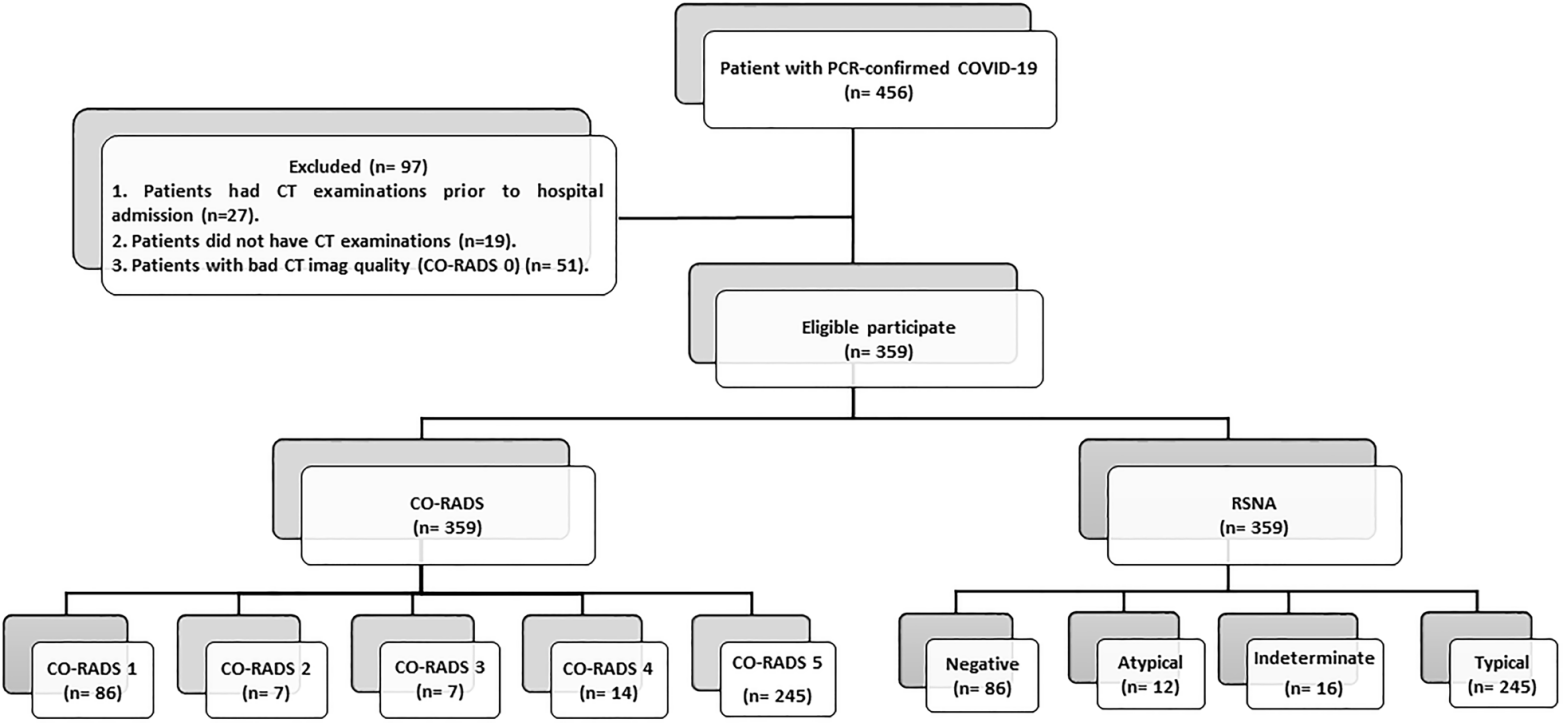
Flowchart of our study

Between April 3, 2020 and May 15, 2020, we identified a total of 456 consecutively admitted patients with swab-confirmed COVID-19 in Assiut University Hospital. Eligible patients included those with swab-confirmed COVID-19 who underwent CT imaging of the chest within 12 h after admission, while the following were grounds for exclusion: (1) CT imaging performed prior to hospital admission (*n* = 27), (2) no CT imaging performed (*n* = 19), and (3) poor CT image quality (*n* = 51). The exclusion process resulted in a final sample consisted of 359 patients. The flowchart of our study population inclusion process is illustrated in Fig. 1. Our participants were classified into three groups based on disease severity as follows: the asymptomatic/mild group included patients with no symptoms or with mild symptoms and no imaging findings of pneumonia; the moderate group included patients with fever, respiratory symptoms, and imaging findings of pneumonia; and the severe/critically ill group included distressed patients with low oxygen saturation (SpO_2_ < 93% at rest) with or without the need for mechanical ventilation or patients in shock or with extrapulmonary organ failure necessitating intensive care unit admission [17].

### CT imaging

All CT scans were performed within one week of RT-PCR. CT imaging was performed using a 16-channel CT scanner (Aquilion Lightning; Toshiba Medical Systems, Tokyo, Japan). No contrast material was administered. Patients were scanned in the supine position, during breath-hold on full inspiration, from the lung apices down to the lung bases. The scanning parameters were as follows: tube voltage, 120 kV; tube current, 50 mA; rotation time, 0.5 s; slice thickness, 5 mm; matrix, 512 × 512; and breath-holding on full inspiration. The protocol was modified in pediatric patients (80 kV and 60 mAs). Reconstruction was carried out in the axial plane with a 1.0-mm slice thickness and 1.0-mm slice interval.

### CT image analysis

All CT images were extracted from the Picture Archiving and Communication Systems and imported into a dedicated workstation (Vitrea® Advanced Visualization; Vital Images, Minnetonka, MN, USA) for image analysis. Three experienced radiologists (M.A, H.M.I, and S.H, with more than 10 years of experience in chest imaging) independently reviewed all CT images. They were blinded to previous CT reports as well as patients’ clinical data, but knew that all patients in the study were COVID-19-positives. Before the beginning of the study, the reviewers were provided with lecture-based and hands-on training that explained the CO-RADS and RSNA chest CT classification systems in detail. The CO-RADS includes five grades as follows: grade 5, very high level of suspicion; grade 4, high level of suspicion; grade 3, equivocal findings; grade 2, low level of suspicion; and grade 1: very low level of suspicion [15]. The CO-RADS grades are further illustrated in Table 1. The RSNA chest CT classification system includes four categories: typical, indeterminate, atypical, and negative (Table 2) [14].

**Table 1:** CO-RADS categories

CO-RADS	Level of suspicion	CT findings
CO-RADS 1	Very low	Normal or non-infectious, e.g. emphysema
CO-RADS 2	Low	Findings consistent with infections other than COVID-19, e.g. tree-in-bud
CO-RADS 3	Equivocal	Equivocal findings for pulmonary involvement of COVID-19 based on CT features that can also be found in other viral pneumonia or non-infectious causes, e.g
		Perihilar GGO
		Homogenous extensive GGO with or without sparing of some secondary pulmonary lobules
		GGO together with smooth interlobular septal thickening with or without pleural effusion in the absence of other typical CT findings
		Small GGO that are not centrilobular or not located close to the visceral pleura
		Patterns of consolidation compatible with organizing pneumonia without other typical findings of COVID-19
CO-RADS 4	High	Findings suspicious for COVID-19 but not typical, e.g. unilateral GGO, multifocal consolidations only
CO-RADS 5	Very high	Typical for COVID-19, e.g. multifocal GGO, peripheral and basal distribution, GGO and consolidations, crazy paving, reversed halo, spider web sign

*CO-RADS* COVID-19 Reporting and Data System, *CT* computed tomography, *COVID-19* coronavirus disease 2019, *GGO* ground-glass opacities

**Table 2:** RSNA chest CT classification system related to COVID-19

RSNA category	CT findings
Typical appearance	Peripheral, bilateral ground-glass opacities (GGO) ± consolidation or visible intralobular lines “crazy paving”
	Multifocal rounded GGO ± consolidation or visible intralobular lines “crazy paving”
	Reverse halo sign
Indeterminate appearance	Absence of typical CT findings and the presence of:
	Multifocal, diffuse, perihilar, or unilateral GGO ± rounded or non-peripheral consolidation
	Few very small GGO with a non-rounded and non-peripheral distribution
Atypical appearance	Absence of above features and the presence of:
	Isolated lobar or segmental consolidation without GGO
	Discrete small nodules e.g. tree-in-bud
	Lung cavitation
	Smooth interlobular septal thickening with pleural effusion
Negative for pneumonia	No GGO and consolidation

*RSNA* radiological Society of North America, *CT* computed tomography, *COVID-19* coronavirus disease 2019, *GGO* ground-glass opacities

The radiologists categorized the CT images of each patient according to the two classification systems at two different times with a one-month interval in between to diminish the radiologists’ memory bias. After independent categorization, inter- and intra-reviewer agreements were evaluated. In the case of a disagreement between reviewers, all parameters were discussed in detail until a final agreement could be reached at least 2 weeks after the second interpretation. The results of the consensus review were used to calculate the sensitivity of both systems.

### Statistical analysis

Categorical variables are represented as numbers and percentages, and the statistical significance was calculated using Chi-squared or Fisher’s exact tests. Continuous data were expressed in the format of mean ± standard deviation. RT-PCR was used as a reference standard for calculating the sensitivity of CT for each reviewer; however, as we did not include cases with negative RT-PCT findings, specificity and predictive values were not calculated. The overall agreement was analyzed using the Fleiss kappa (*κ*) test. The *κ* values were interpreted as follows: 0–0.2, no agreement; 0.21–0.4, weak agreement; 0.41–0.60, moderate agreement; 0.61–0.80, good agreement; and 0.81–1.0, excellent agreement. Inter-reviewer agreement of the categories of each system was defined by the use of an intraclass correlation coefficient. Statistical analysis was carried out using the Statistical Package for the Social Sciences version 26 (IBM Corporation, Armonk, NY, USA). Statistical significance was defined as *p* < 0.05.

## Results

### Patient characteristics

The final analysis included a total of 359 patients (180 men, 179 women; mean age, 45 ± 16.9 years; range, 1–90 years) who were COVID-19-positive confirmed by RT-PCR. The study participants’data are summarized in Table 3. With respect to disease severity, 96 (26.7%) patients were asymptomatic/had mild disease, 171 (47.6%) patients had moderate disease, and 92 (25.6%) patients had severe disease/were critically ill. Death occurred in 22 (6.1%) patients; all were categorized with a CO-RADS 5 and typical RSNA classification.

**Table 3:** Baseline demographics and clinical characteristics of study participants stratified by clinical category

Characteristic	All (*n* = 359)	Mild (*n* = 96)	Moderate (*n* = 171)	Severe (*n* = 92)	*p* value
Age, mean ± SD	45 ± 16.9	30.7 ± 14.2	48.6 ± 15.2	53.5 ± 13.1	0.000
Gender					0.045
Male	180 (50.1)	39 (40.6)	87 (50.9)	54 (58.7)	
Female	179 (49.9)	57 (59.4)	84 (49.1)	38 (41.3)	
Symptoms					
Fever	273 (76)	41 (42.7)	142 (83.0)	90 (97.8)	0.000
Cough	259 (72.1)	37 (38.5)	135 (78.9)	87 (94.5)	0.000
DOB	202 (56.3)	0 (0)	123 (71.9)	79 (85.9)	0.000
Diarrhea	20 (5.6)	5 (5.2)	8 (4.6)	7 (7.6)	0.512
Comorbidities	63 (17.5)	10 (10.4)	25 (14.6)	28 (30.4)	0.001
Hypertension	24 (6.7)	5 (5.2)	10 (5.8)	9 (9.8)	0.379
Diabetes	15 (4.2)	2 (2.1)	5 (2.9)	8 (8.7)	0.041
COPD	11 (3.1)	1 (1.0)	4 (2.3)	6 (6.5)	0.070
Heart disease	9 (2.5)	2 (2.1)	6 (3.5)	1 (1.1)	0.456
CKD	4 (1.1)	0 (0)	0 (0)	4 (4.3)	0.004
Death	22 (6.1)	0 (0)	0 (0)	22 (23.7)	0.000

Unless otherwise indicated, date represent the number of patients and percentage in parentheses

*n* number, *SD* standard deviation, *DOB* difficulty of breathing, *COPD* Chronic obstructive pulmonary disease, *CKD* Chronic kidney disease

### Assignment of CO-RADS and RSNA chest CT classification system categories

The categorization of patients based on CO-RADS and the RSNA chest CT classification system with regard to age is presented in Table 4. A highly statistically significant relationship was found between CT findings and age group (*p* < 0.001). Disease of CO-RADS 5 and typical RSNA classification was more commonly recorded among those aged older than 50 years (88.6%). On the other hand, patients younger than 15 years totaled only 2.5% of all participants and none had disease of CO-RADS 5 or the typical RSNA category; only one two-year-old child presented with disease of CO-RADS 4 and the indeterminate RSNA category.

**Table 4:** Assignment of CO-RADS and the RSNA chest CT classification system categories in relation to patient age

	< 15 years (*n* = 9)	15–50 years (*n* = 192)	> 50 years (*n* = 158)	Total (*n* = 359)
CO-RADS				
CO-RADS 1	8 (88.9)	69 (35.9)	9 (5.7)	86 (24.0)
CO-RADS 2	0 (0)	4 (2.1)	3 (1.9)	7 (1.9)
CO-RADS 3	0 (0)	5 (2.6)	2 (1.3)	7 (1.9)
CO-RADS 4	1 (11.1)	9 (4.7)	4 (2.5)	14 (3.9)
CO-RADS 5	0 (0)	105 (54.7)	140 (88.6)	245 (68.2)
RSNA system				
Negative	8 (88.9)	69 (35.9)	9 (5.7)	86 (24.0)
Atypical	0 (0)	7 (3.6)	5 (3.2)	12 (3.3)
Indeterminate	1 (11.1)	11 (5.7)	4 (2.5)	16 (4.5)
Typical pattern	0 (0)	105 (54.7)	140 (88.6)	245 (68.2)

Date represent the number of patients and percentage in parenthesis

*n* number, *CO-RADS* COVID-19 Reporting and Data System, *RSNA* radiological Society of North America, *CT* computed tomography, *COVID-19* coronavirus disease 2019

### The sensitivity of each classification system

Considering combined CO-RADS 3, 4 and 5 as a positive predictor for COVID-19 diagnosis, the sensitivity of CO-RADS was 9.4%, 94.7%, and 97.8%, in the asymptomatic/mild disease group, moderate disease group, and severe/critically ill disease group, respectively. Similar sensitivities were found when considering the typical and indeterminate RSNA categories together as a positive predictor for COVID-19 diagnosis (9.4%, 94.7%, and 97.8% in the asymptomatic/mild disease group, moderate disease group, and severe/critically ill disease group, respectively) (Table 5).

**Table 5:** Sensitivity of CO-RADS and the RSNA chest CT classification system in the diagnosis of COVID-19 patients stratified by clinical group

	Cut-off value	True-positive	False-negative	Sensitivity
CO-RADS	≥ CO-RADS 3			
Asymptomatic/mild group (*n* = 96)		9	87	9.4%
Moderate group (*n* = 171)		162	9	94.7%
Severe/critically ill group (*n* = 92)		90	2	97.8%
Overall (*n* = 359)		261	98	72.7%
RSNA	Typical + indeterminate patterns			
Asymptomatic/mild (*n* = 96)		9	87	9.4%
Moderate (*n* = 171)		162	9	94.7%
Severe/critical group (*n* = 92)		90	2	97.8%
Overall (*n* = 359)		261	98	72.7%

*n* number, *COVID-19* coronavirus disease 2019, *RSN*A radiological Society of North America, *CO-RADS* COVID-19 Reporting and Data System

### The reliability of each classification system

Table 6 shows the inter-reviewer agreement for the two classification systems stratified according to different categories. Among the reviewers, the overall inter-reviewer agreement was excellent for CO-RADS (*κ* = 0.801) and good for the RSNA chest CT classification system (*κ* = 0.781). Separately, the inter-reviewer agreement for individual diagnostic categories was excellent for CO-RAD 1 (*κ* = 0.924),CO-RAD-5 (*κ* = 0.888), the negative RSNA category (*κ* = 0.924), and the typical RSNA category (*κ* = 0.841); moderate for CO-RAD 4 (*κ* = 0.463); and weak for CO-RAD 2 (*κ* = 0.303), the indeterminate RSNA category (*κ* = 0.386), and the atypical RSNA category (*κ* = 0.380). CO-RAD 3 showed no agreement (*κ* =  −0.017).

**Table 6:** Inter-reviewer agreement for CO-RADS and the RSNA chest CT classification system stratified according to different categories

Rating category	Kappa	95% CI
CO-RADS		
CO-RADS 1	0.930	(0.928 to 0.931)
CO-RADS 2	0.303	(0.301 to0.305)
CO-RADS 3	− 0.018	(− 0.020 to − 0.016)
CO-RADS 4	0.464	(0.462 to 0.465)
CO-RADS 5	0.888	(0.886 to 0.890)
Overall	0.801	(0.800 to 0.803)
RSNA		
Negative	0.924	(0.922 to 0.926)
Atypical	0.380	(0.378 to 0.382)
Indeterminate	0.386	(0.384 to 0.388)
Typical	0.841	(0.839 to 0.843)
Overall	0.781	(0.780 to 0.782

*CO-RADS* COVID-19 Reporting and Data System, *RSNA* radiological Society of North America, *CT* computed tomography, *CI* confidence interval

Table 7 shows the intra-reviewer agreement for the two systems stratified according to different categories and reviewers. The intra-reviewer agreement was excellent for all reviewers for CO-RADS 1, and 5, and the negative RSNA, and typical RSNA categories and good to excellent for CO-RADS 4 and the indeterminate RSNA category. No agreement was found for CO-RADS 2, or 3, or the atypical RSNA category.

**Table 7:** Intra-reviewer agreement for CO-RADS and the RSNA chest CT classification system stratified according to different categories and reviewers

Rating category	Reviewer 1	Reviewer 2	Reviewer 3
CO-RADS			
CO-RADS 1	1.000	0.959	1.000
CO-RADS 2	− 0.007	− 0.007	0.000
CO-RADS 3	− 0.014	− 0.007	− 0.007
CO-RADS 4	0.850	0.793	0.735
CO-RADS 5	1.000	1.000	0.893
Overall	0.933	0.932	0.900
RSNA			
Negative	0.915	0.959	1.000
Atypical	− 0.007	− 0.007	0.000
Indeterminate	0.850	1.000	0.704
Typical	1.000	1.000	0.897
Overall	0.932	0.966	0.904

*CO-RADS* COVID-19 Reporting and Data System, *RSNA* radiological Society of North America, *CT* computed tomography, *CI* confidence interval

**Fig. 2:**
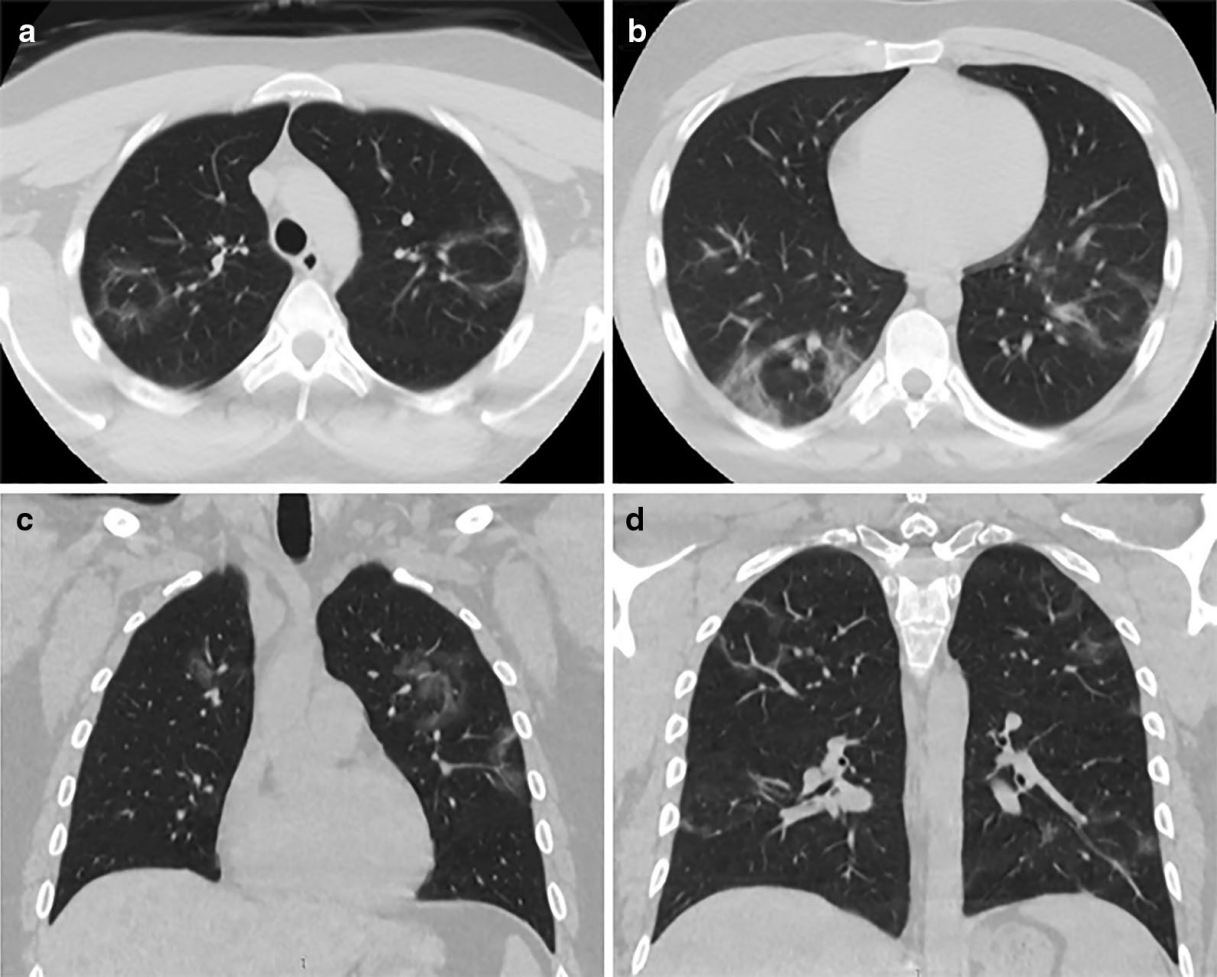
A 28-year-old man with positive RT-PCR findings for COVID-19. **a**–**d** Noncontrast axial CT images of the chest show bilateral peripheral ground-glass opacities, bilateral reverse halo sign, and prominent vessel inside. This is in keeping with his CO-RADS 5 and typical RSNA classification

**Fig. 3:**
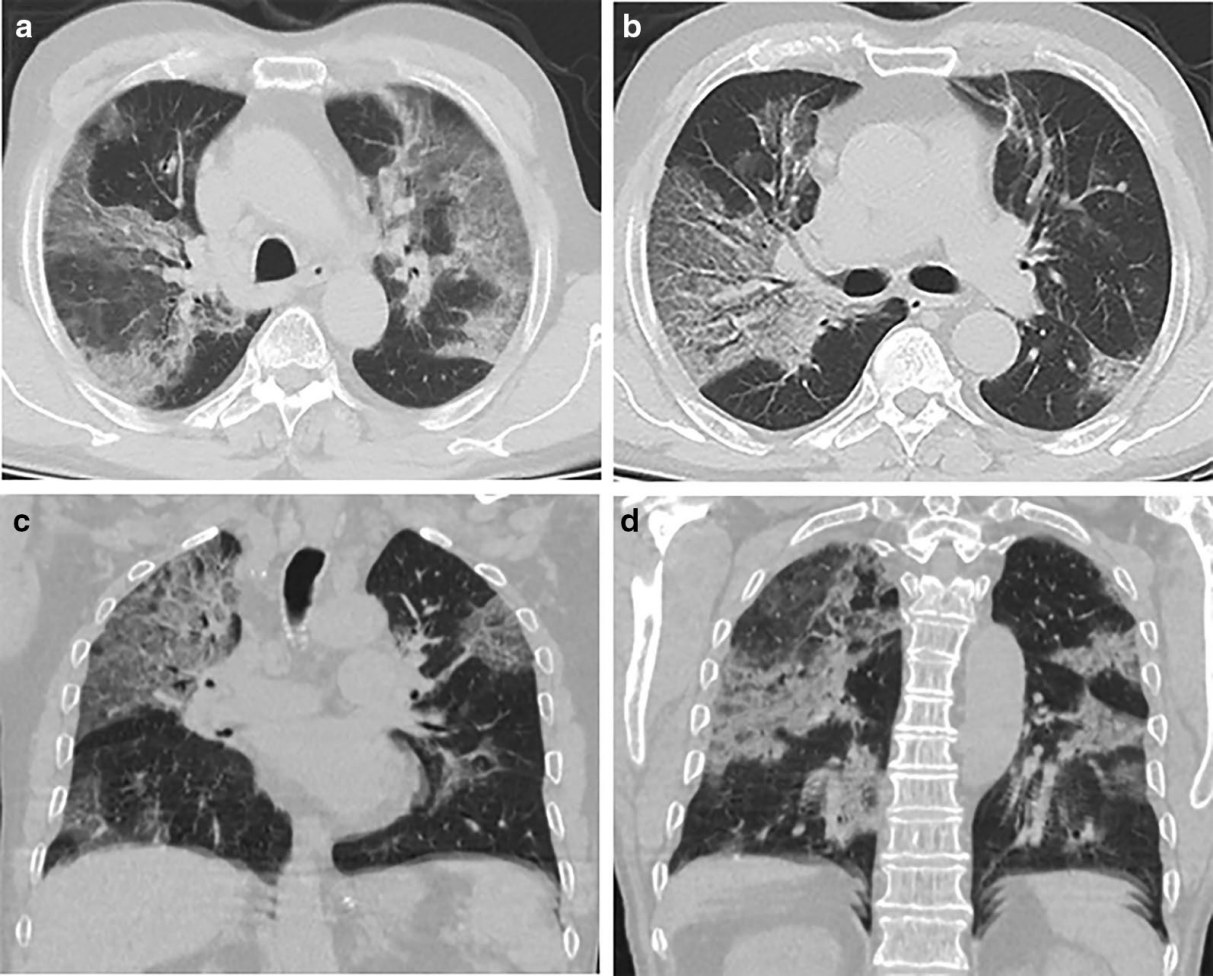
A 67-year-old man with positive RT-PCR findings for COVID-19. **a–d** Noncontrast axial CT images of the chest show bilateral, multifocal peripheral ground-glass opacities with superimposed interlobular septal thickening and intralobular lines are visible, giving the appearance of “crazy-paving”. This is in keeping with his CO-RADS 5 and typical RSNA classification

**Fig. 4:**
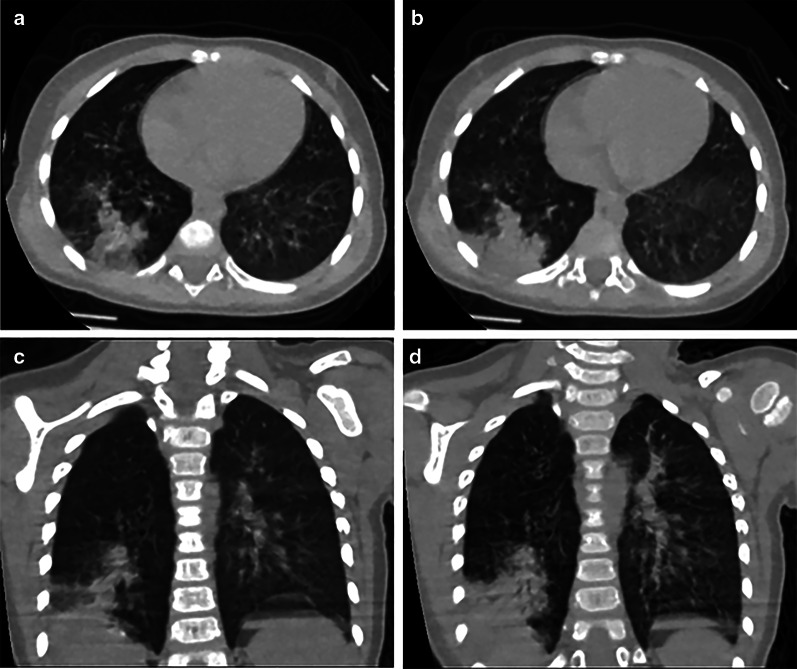
A two-year-old male child with positive RT-PCR findings for COVID-19. **a**–**d** Noncontrast axial CT images of the chest show right lower-lobe peripheral consolidation and surrounding ground-glass opacity. This is in keeping with his CO-RADS 4 and indeterminate RSNA classification

Representative cases from our study are illustrated in Figs. 2, 3 and 4.

## Discussion

The diagnosis of COVID-19, especially mild forms of the disease, constitutes one of the major challenges in clinical practice nowadays. CO-RADS and the RSNA chest CT classification system are the results of efforts made to create a uniform CT-based classification for the diagnosis of COVID-19. However, global studies of these classification systems are still limited in number. The current study is an attempt to assess and compare the sensitivity and reliability of these two systems.

The overall results demonstrated that both systems are comparable to one another, with similar sensitivity values. Considering the combination of CO-RADS 3, 4 and 5 and the combination of the typical and indeterminate RSNA categories, respectively, as positive predictors for COVID-19 diagnosis, the overall sensitivity was the same for both classification systems (72.7%). Meanwhile, the sensitivity significantly increased for both systems when excluding the asymptomatic/mild patients and considering only moderate (sensitivity = 94.7%) and severe/critically ill patients (sensitivity = 97.8%); this is not surprising, taking into account that the sensitivity depends on CT imaging features, which have been considerably proven in several recent studies [6, 18–22], while CT has been confirmed to be a reliable imaging approach for the evaluation of COVID-19. Our data are congruent with the results mentioned in previous research [15, 23–28], which suggested that the CO-RADS and the RSNA chest CT classification system performed very well in predicting COVID-19 in patients with moderate to severe symptoms. Notably, a recent meta-analysis published by Kwee et al. [29] concluded that COVID-19 infection frequency was higher in patients categorized with higher CO-RADS and RSNA classification categories.

A remarkable finding in our study was the high proportion of false-negative results (*n* = 100 patients; 27.9%); of these, 98 patients were categorized as CO-RADS 1 and 2 and RSNA classification categories negative and a typical. This high proportion of false-negative results was due to the fact that CT chest imaging was performed early in the disease course. Comparable results were reported by Prokop et al. [15] and Bernheim et al. [30].

Although higher categories had high sensitivity for both classification systems, false-negative results were high, too. Therefore, lower categories could not exclude COVID-19. These results agree with the recent meta-analysis published by Kwee et al. [29], which reported that CO-RADS 1 and 2 and RSNA classification categories negative and a typical do not exclude COVID-19.

The reliability is crucial for evaluating a new classification system. An analysis of our results demonstrated that CO-RADS and the RSNA chest CT classification system had comparable overall good to excellent inter-reviewer agreement, with a higher level of agreement achieved for CO-RADS (*κ* = 0.801) than for the RSNA chest CT classification system (*κ* = 0.781). Meanwhile, the intra-reviewer agreement was excellent for both systems, although it tended to be lower for CO-RADS 2 and 3 and for the indeterminate and atypical RSNA categories. The reason for the lower agreement in the intermediate categories of both systems may be largely related to the fact that all the patients were actually COVID-19-positives, while those categories are meant for alternative diagnoses. However, if there were real lobar pneumonias or "tree in bud" patterns, the agreement would have been higher. Our results are in line with those of several previous studies [15, 23, 28, 31, 32]. Prokop et al. [15] conducted the first study that investigated the consistency of CO-RADS and reported a reasonable level of moderate intra-reviewer agreement (*κ* = 0.47), with the highest agreement noted for CO-RADS 1 (*κ* = 0.58) and 5 (*κ* = 0.68). A recent study published by Bellini et al. [23] indicated a moderate level of overall agreement was obtained for CO-RADS (*κ* = 0.43), with less agreement achieved for the intermediate (grades 2–4) CO-RADS categories than for CO-RADS 1 and 5. Separately, in a study conducted by Ciccarese et al. [28], two readers evaluated 460 patients according to the RSNA chest CT classification system and achieved a good level of inter-reviewer agreement for the typical and negative categories and a fair level of inter-reviewer agreement for the indeterminate and atypical categories (*κ* = 0.5). Another study [31] investigated inter-reviewer agreement for the RSNA chest CT classification system and reported excellent agreement for typical, atypical, and negative RSNA categories and good agreement for the indeterminate category. A more recent study published by Inui et al. [32] reported good inter-reviewer agreement for CO-RADS (*κ* = 0.62) and the RSNA classification (*κ* = 0.63).

Regarding patient demographics, we found that pulmonary changes are less likely to occur at a young age. Among nine study participants younger than 15 years, only one patient, a two-year-old male child, developed pneumonia (CO-RADS 4 and indeterminate RSNA category). This finding is unsurprising given that COVID-19 has a predominantly mild presentation and a good prognosis in children, with rare occurrences of death. A study published by Zheng et al. [33] concluded that children with COVID-19 similarly had a more favorable clinical presentation than adults; however, those younger than three years old were more susceptible to developing severe illness. In our study, most adults presented with high CO-RADS grades and the typical RSNA category. This finding agrees with the well-known conclusion that old age is a predisposing factor for COVID-19 pneumonia [34]. An interesting finding in our study was that all death cases occurred in patients with CO-RADS 5 and the typical RSNA category. This finding might reassure some about the patient outcome for those with results below CO-RADS 5 and the typical RSNA category. However, identifying those with lower scores is still important in facilitating the isolation of infected patients.

In summary, based on these findings, which resemble those of the aforementioned published studies, we found that both systems are comparable, with similar sensitivity and reliability values, and suggest that using either system will yield the same results. Along these lines, both systems performed well when applied in moderate and severe/critically ill patients. However, some limitations are present in our study. First, this study was retrospective and performed in a single center. Second, our study included only the first CT chest performed around the time of admission irrespective of the number of days that had elapsed since the appearance of symptoms. Third, the specificity and predictive values of CO-RADS and the RSNA chest CT classification system for the diagnosis of COVID-19 were not established, as we did not include COVID-19 negative-patients in our analysis. Fourth, we did not consider the impact of comorbidity factors on the sensitivity of CO-RADS and RSNA classification. Fifth, the radiologists who reviewed the CT images known that all patients participating in the study were positive for COVID-19, which may be a source of bias. Finally, many clinicians are still unfamiliar with the CO-RADS and RSNA CT classification system and might misunderstand these schemes as simple indicators of disease severity unless the CT-severity score is stated in the report.

## Conclusion

In conclusion, our results support that CO-RADS and the RSNA chest CT classification systems are comparable to one another in the diagnosis of COVID-19 pneumonia with similar sensitivity and reliability values. Applying these systems in patients with moderate and severe symptoms will significantly improve their sensitivity for diagnosing COVID-19 pneumonia. However, CO-RADS had a better inter-reviewer agreement that may be attributed to greater familiarity with the CO-RADS system among radiologists due to its resemblance to other RAD systems.

## Data Availability

The datasets used and/or analyzed during the current study are available from the corresponding author on reasonable request.

## Abbreviations

CO-RADS: Coronavirus disease 2019 reporting and data system
COVID-19: Coronavirus disease 2019
CT: Computed tomography
GGO: Ground-glass opacities
ICC: Intraclass correlation coefficien
IRB: Institutional review boards
RSNA: Radiological Society of North America
RT-PCR: Real-time reverse-transcriptase-polymerase chain reaction
SARS: Severe acute respiratory syndrome

## Declarations

### Statistics and biometry

The corresponding author has great statistical expertise.

### Methodology

(1) Retrospective; (2) Diagnostic or prognostic study; (3) Performed at single centre.
